# CF-DETR: A Lightweight Real-Time Model for Chicken Face Detection in High-Density Poultry Farming

**DOI:** 10.3390/ani15192919

**Published:** 2025-10-08

**Authors:** Bin Gao, Wanchao Zhang, Deqi Hao, Kaisi Yang, Changxi Chen

**Affiliations:** 1 Key Laboratory of Smart Breeding (Co-Construction by Ministry and Province), Ministry of Agriculture and Rural Affairs, Tianjin 300384, China; bingao_1594@163.com (B.G.); 163109306@stud.tjut.edu.cn (K.Y.); 2 College of Computer and Information Engineering, Tianjin Agricultural University, Tianjin 300384, China; baiduziliao2@163.com (W.Z.); haodeqi_3178@163.com (D.H.)

**Keywords:** chicken face detection, CF-DETR, real-time detection, precision poultry

## Abstract

**Simple Summary:**

Modern poultry farms often use automated systems to monitor flock health, but overlapping chickens and changing lighting make individual face detection challenging. Traditional methods struggle to identify each bird reliably in these conditions. In this study, we present CF-DETR, a new deep-learning model that detects chicken faces in real time. The model uses special computer vision techniques to capture details even when birds overlap or move. We tested CF-DETR on images from crowded farm environments. It detected over 95% of chicken faces correctly and processed video at around 80 frames per second. These results show CF-DETR can robustly monitor chickens in dense flocks under challenging conditions. This automatic monitoring system could help farmers track poultry health earlier and more efficiently, improving animal welfare and farm productivity. By accurately detecting each chicken’s face, CF-DETR could help farmers spot signs of disease or stress earlier. Such smart monitoring is valuable for modern intelligent poultry farming.

**Abstract:**

Reliable individual detection under dense and cluttered conditions is a prerequisite for automated monitoring in modern poultry systems. We propose CF-DETR, an end-to-end detector that builds on RT-DETR and is tailored to chicken face detection in production-like environments. CF-DETR advances three technical directions: Dynamic Inception Depthwise Convolution (DIDC) expands directional and multi-scale receptive fields while remaining lightweight, Polar Embedded Multi-Scale Encoder (PEMD) restores global context and fuses multi-scale information to compensate for lost high-frequency details, and a Matchability Aware Loss (MAL) aligns predicted confidence with localization quality to accelerate convergence and improve discrimination. On a comprehensive broiler dataset, CF-DETR achieves a mean average precision at IoU 0.50 of 96.9% and a mean average precision (IoU 0.50–0.95) of 62.8%. Compared to the RT-DETR baseline, CF-DETR reduces trainable parameters by 33.2% and lowers FLOPs by 23.0% while achieving 81.4 frames per second. Ablation studies confirm that each module contributes to performance gains and that the combined design materially enhances robustness to occlusion and background clutter. Owing to its lightweight design, CF-DETR is well-suited for deployment in real-time smart farming monitoring systems. These results indicate that CF-DETR delivers an improved trade-off between detection performance and computational cost for real-time visual monitoring in intensive poultry production.

## 1. Introduction

Broiler production plays a pivotal role in the global supply of animal protein. With continued population growth and accelerated urbanization, broiler chickens have become a primary source of animal protein because of their high productivity and low production cost [1]. The broiler industry not only contributes to food security and market supply but also plays an important role in promoting agricultural economic development and increasing farmers’ incomes [2,3]. In recent years, driven by rapid expansion of farming scale and growing demand for intelligent management, precision poultry farming has become a major development direction [4]. This approach integrates modern sensing and information technologies to enable automated and fine-grained monitoring of flocks, aiming to improve production efficiency while safeguarding animal welfare [5].

In precision farming scenarios, comprehensive collection of individual level information supports the formulation of scientific breeding strategies, enables early disease warning, and effectively reduces systemic risks during production [4,6,7]. Precise detection of individual broilers within a flock is the essential first step for acquiring such detailed individual information [8]. Previous studies have shown that the facial region of chickens exhibits relatively stable texture and morphological patterns, and therefore it serves as a key anatomical site for individual identification and health monitoring [8,9]. Accordingly, chicken face detection not only provides a feasible pathway for identity recognition but also constitutes a core component for building intelligent visual perception systems.

However, high density rearing environments are often characterized by severe occlusion among individuals, frequent pose variation, and complex illumination conditions [10], which pose substantial challenges to chicken face detection [11,12]. At the same time, traditional manual observation and video replay methods are inefficient and costly, and they struggle to meet the real-time and large-scale processing requirements of modern farms [13]. These limitations have accelerated the adoption of deep learning-based object detection techniques for avian monitoring.

In the object detection field, the YOLO series has been widely applied to poultry monitoring because of its favorable trade off between speed and accuracy [14,15]. Some studies have employed YOLO-based convolutional neural networks to detect broiler heads and to estimate feeding duration by detecting head entry into feeders [16]. Another study built a broiler leg disease detection model using YOLOv8 and demonstrated promising performance for early lesion diagnosis [17]. To address dense occlusion, researchers have proposed single class dense detection networks such as YOLO-SDD to enhance robustness [11]. Other work has applied YOLOv5 for diseased bird detection and reported an accuracy of approximately 89.2 percent [18]. Nevertheless, these YOLO-based detectors typically rely on non-maximum suppression NMS for post processing, which reduces inference speed and introduces multiple hyperparameters, thus causing instability in the speed versus accuracy trade off [14].

By contrast, DETR, the DEtection TRansformer, employs a Transformer architecture and a set prediction mechanism to form an end-to-end detection pipeline and thereby eliminates dependence on NMS [19]. However, despite removing post processing, the standard DETR design incurs considerable computational overhead and its inference speed often fails to meet real time requirements [19]. To this end, RT-DETR retains the advantages of the DETR paradigm while introducing a convolutional backbone and an efficient hybrid encoder, achieving a more favorable balance between detection accuracy and speed [20].

Although RT-DETR demonstrates advantages in structural simplicity and global modeling capability, its original ResNet backbone has limited representational power when handling targets with highly similar structures and subtle texture variations, making it difficult to capture fine interindividual differences [21]. In addition, the built-in Anchor Free Instance Interaction AIFI architecture exhibits insufficient synergy between global modeling and local detail recovery in the chicken face detection context, and normalization procedures show degraded efficiency under parallel computation. These issues are accompanied by high computational and memory costs, loss of fine detail information, and inconsistent behavior across scales [22,23,24,25]. Meanwhile, the Varifocal Loss VFL used in RT-DETR is insensitive to low-quality matching samples, which results in insufficient discrimination between positive and negative samples and thereby limits the effectiveness of matching optimization and the speed of training convergence [26].

In summary, although RT-DETR offers end-to-end detection benefits, it still faces multiple bottlenecks in backbone design, encoding mechanisms, and loss formulation, which hinder its direct application to chicken face detection, a task that involves complex structural patterns and subtle textures. To meet the accuracy and real-time requirements of chicken face detection in complex farm environments, this paper proposes a systematic set of structural optimizations to the RT-DETR framework that focuses on three critical aspects of the detection pipeline: feature extraction, information encoding, and loss design. By synergistically improving these components, we construct an end-to-end detection model CF-DETR with enhanced structural adaptability and expressive capacity. The main contributions are summarized as follows:

(i) Dynamic Inception Depthwise Convolution DIDC. To overcome limitations of conventional backbones in representing diverse spatial structures, we design a convolutional operator based on multi path depthwise separable convolutions and a dynamic fusion mechanism to strengthen feature extraction under complex poses and occlusion.
(ii) Polar Embedded Multi-scale Encoder PEMD. To address key issues of global modeling, normalization efficiency, multi-scale fusion, and detail compensation, we propose a structurally closed loop encoder module that improves coordination and stability in representing global context and local texture.
(iii) Matchability Aware Loss MAL. In the design of training objectives, we introduce a dynamic label weighting mechanism, so that predicted confidence adapts to matching strength, thereby optimizing training convergence and enhancing discriminability between targets and background.

## 2. Materials and Methods

### 2.1. Image Acquisition

**Figure 1:**
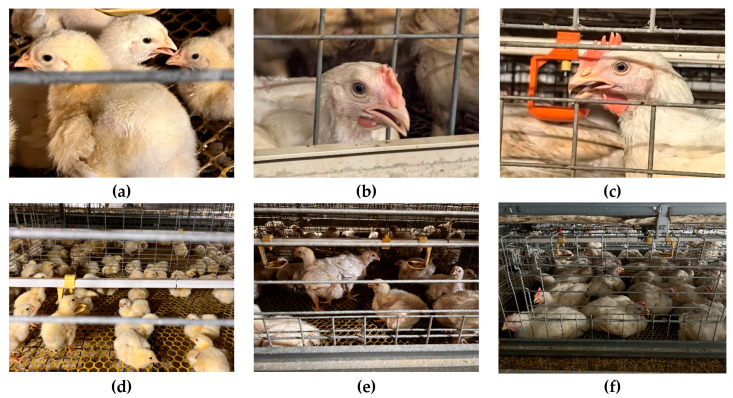
Facial images of broilers at different growth stages: panels (**a**,**d**) correspond to the starter period, panels (**b**,**e**) correspond to the grower period, and panels (**c**,**f**) correspond to the finisher period.

Broiler growth can be divided into three stages: the starter period from 0 to 14 days, the grower period from 15 to 28 days, and the finisher period from day 29 until slaughter. During the starter period the facial region of chickens is small and exhibits low discriminative features. In the grower period the facial area increases gradually and the discriminability of facial features improves markedly. In the finisher period the facial region is fully developed and shows the greatest stability and discriminative power. To enhance the model performance across the entire production cycle we systematically collected facial images from birds from entry at day of age zero through to the slaughter stage. Samples were obtained from a commercial broiler breeding farm in Xinxing County Yunfu City Guangdong Province and WOD168 white-feather broilers were used as the study subjects. Image acquisition was performed with a full color low light night vision camera module (Shenzhi Weilai; Shenzhen, China) yielding a total of 1000 valid images. All data were split into training validation and test subsets at a ratio of 8:1:1 to ensure rigorous and reliable model training and evaluation. Figure 1 illustrates pronounced differences in facial geometry and texture across the growth stages and thereby reflects the diversity in spatial distribution and appearance of the samples which provides a solid basis for evaluating model robustness and generalization.

### 2.2. Data Annotation and Augmentation

**Figure 2:**
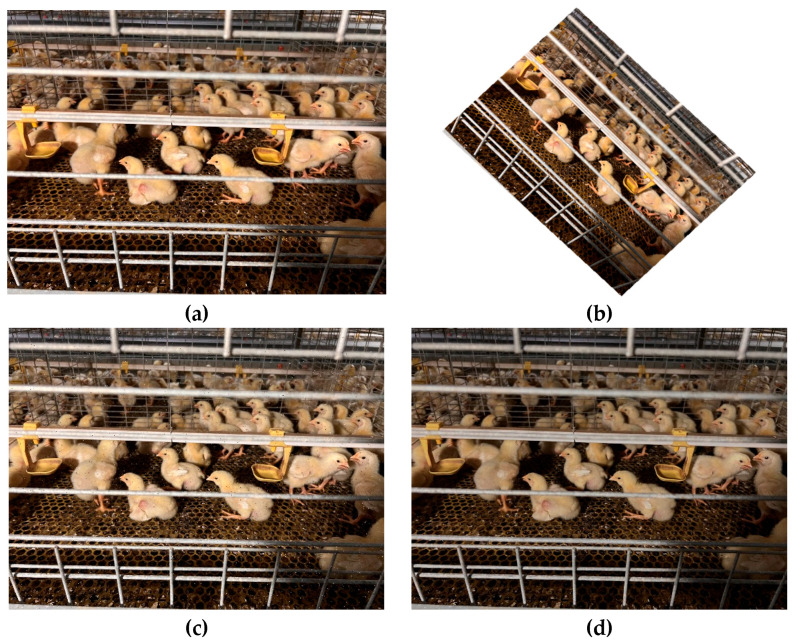
Examples of data augmentation: (**a**) original chicken face image, (**b**) random rotation from minus 45 degrees to 45 degrees, (**c**) injected salt and pepper noise, and (**d**) random gamma correction combined with HSV brightness perturbation.

**Figure 3:**
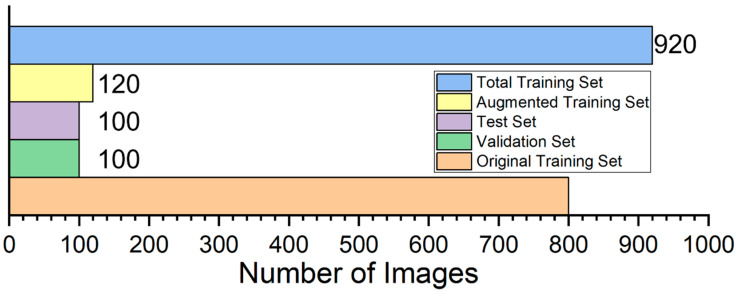
Schematic diagram of the data split for the training set, validation set, and test set.

We first used the LabelImg tool to generate precise annotations of chicken face regions in the training validation and test sets. To address dynamic challenges in real farm environments such as frequent pose variation noise interference and illumination fluctuation we designed and applied three data augmentation strategies to the training set. As shown in Figure 2 the first strategy applies random rotation in the range minus 45 degrees to 45 degrees to simulate natural head movements and to improve the model’s adaptability to diverse poses [27]. The second strategy injects salt and pepper noise at densities between 0.5 percent and 3 percent to simulate lens dust accumulation and sensor aging and thereby strengthen the model’s noise robustness [28]. The third strategy combines random gamma correction with gamma sampled from the interval 0.5 to 1.5 and brightness perturbation in the HSV space by plus or minus 20 percent to mimic illumination changes across different times of day and weather conditions and thus improve illumination generalization [29]. These augmentation operations produced an additional 120 images from the original 800 image training set, expanding the training set to 920 images while the validation and test sets remained at 100 images each. The detailed partitioning of the three subsets is reported in Table 1 and illustrated in Figure 3 ensuring the rigor and comprehensiveness of model training and evaluation.

### 2.3. Construction of the Chicken Face Detection Model

#### 2.3.1. CF-DETR Model Construction

**Figure 4:**
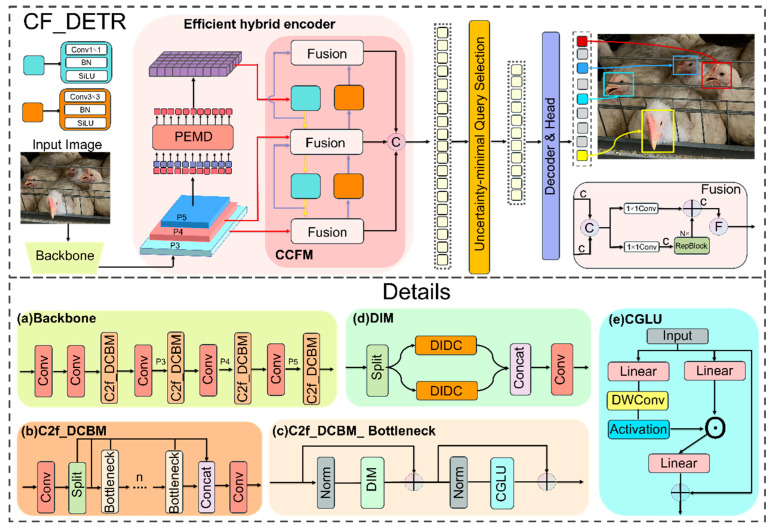
CF-DETR network diagram. Subfigure (**a**) illustrates the overall structure of the proposed improved backbone, which contains the core feature extraction module shown in (**b**). Subfigure (**b**) further includes the basic residual unit presented in (**c**), while subfigure (**c**) is composed of two parts, (**d**,**e**), forming the C2f DCBM bottleneck. The DIDC module displayed in (**d**) is the novel component introduced in this study and is described in detail in Section 2.3.2.

In this study we propose CF-DETR. As shown in Figure 4, the core idea is to perform systematic and collaborative optimization of the following three components: the backbone, the encoder, and the loss function. First, the backbone core module DIDC extracts multi-scale spatial features in parallel and uses global context to adaptively fuse the outputs of each branch thereby enhancing representational capacity under lightweight constraints. At the encoder side the PEMD synthesis module performs four sequential operations. It first employs PolaAttention to efficiently reconstruct global dependencies in the polar coordinate domain; then, it uses DynamicTanh to perform smooth channel normalization and accelerate computation; next, it relies on the Mona submodule to juxtapose multi-receptive field branches for collaborative aggregation of local and global information; and finally, it applies EDFFN for directional compensation of high frequency details so that a closed loop feature flow is formed between global context and detail recovery. Lastly, the Matchability Aware Loss (MAL) replaces conventional classification targets with matchability aware labels that impose stronger penalties on low quality predictions as confidence increases, thereby accelerating training convergence and improving localization accuracy. Overall, CF-DETR integrates these three modules to construct a lighter and more efficient end-to-end chicken face detection framework that provides solid support for real-time high accuracy detection in complex farming environments.

#### 2.3.2. DIDC Module

**Figure 5:**
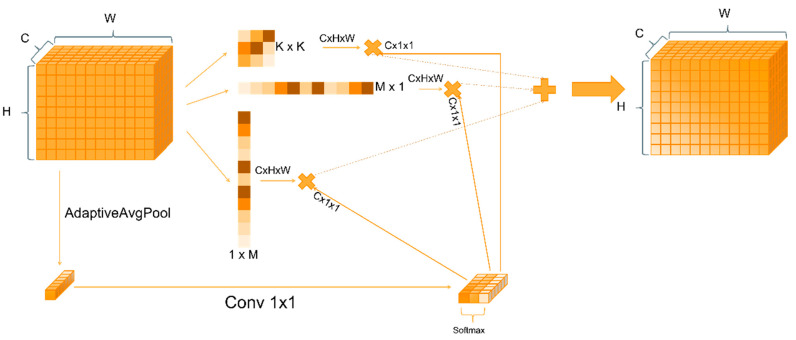
Structure diagram of the DIDC module. DIDC comprises three parallel depthwise separable convolution branches, a square branch, a horizontal strip branch, and a vertical strip branch, which extract spatial features across different orientations and receptive fields.

To improve detection robustness and structural adaptability of the facial region under complex conditions, we propose a backbone design based on dynamic multi-scale modeling whose core innovation is the Dynamic Inception Depthwise Convolution (DIDC) module. As shown in Figure 5, DIDC employs three parallel depthwise separable convolution paths comprising a square convolution path, a horizontal strip convolution path, and a vertical strip convolution path to extract spatial features with different directions and receptive fields. At the same time, the module generates dynamic weights from global context to achieve adaptive fusion of multi-scale features.

Building on this design we further construct a Dynamic Inception featuring Mixing module (DIM) as detailed in the inset of Figure 4. DIM uses the DIDC module as the central operator and replaces a single depthwise convolution path with a multi-path spatial featuring mixing unit. This unit is embedded in a Transformer-style dual residual structure and works in concert with a gated CGLU mechanism to form the C2f_DCBM_bottleneck. Inspired by the YOLOv8 backbone we then assemble an improved backbone block C2f_DCMB that integrates the above designs and deploys the block throughout the RT-DETR backbone.

In this section we detail the design and mathematical formulation of the DIDC module to elucidate how it adaptively fuses multi-scale spatial features within a lightweight framework. As shown in Figure 5 the operator is built upon the input feature map $V\in R^{B\times C\times H\times W}$ and simultaneously constructs three depthwise separable convolution branches to cover distinct receptive fields:

(1) $$F_{1}\left(V\right)=DWC{\mathrm{onv}}_{k\times k}\left(V\right)$$

(2) $$F_{2}\left(V\right)=DWC{\mathrm{onv}}_{1\times b}\left(V\right)$$

(3) $$F_{3}\left(V\right)=DWC{\mathrm{onv}}_{b\times 1}\left(V\right)$$

F_1_ denotes the square convolution branch, F_2_ denotes the horizontal strip convolution branch, and F_3_ denotes the vertical strip convolution branch. The strip convolution kernel width is set to b=3k+2 to extend the response capability for extreme aspect ratios. The three parallel branches can therefore extract both local compact features and elongated structural features while maintaining high efficiency in a single forward pass.

To allocate each branch’s contribution adaptively according to input content, the operator extracts a weighting signal from the global context. Global average pooling is applied to V to obtain the tensor:

(4) $$U=GAP\left(V\right)\in R^{B\times C\times 1\times 1}$$

It is then mapped to 3C channels by a 1 × 1 convolution.

(5) $$S=Conv_{1\times 1}\left(U\right)\in R^{B\times 3C\times 1\times 1}$$

S is reshaped along the channel axis into three segments ${\left\{S_{i}\right\}}_{i=1}^{3}$, and Softmax normalization is applied along the branch dimension to obtain the dynamic weights:

(6) $$\alpha_{i}=\frac{\exp\left(S_{i}\right)}{\sum_{j=1}^{3}\exp\left(s_{j}\right)},\;i=1,2,3.$$

This mechanism enables each convolutional branch at different scales to incorporate global semantic information and adaptively adjust the importance of its output during every forward computation. Finally, the weighted branch outputs are aggregated and subsequently passed through batch normalization and SiLU activation to generate the final feature map:

(7) $$Y=SiLU\left(BN\left(\alpha_{1}F_{1}\left(V\right)+\alpha_{2}F_{2}\left(V\right)+\alpha_{3}F_{3}\left(V\right)\right)\right)$$

In summary, the C2f_DCMB module, with DIMC as its core, leverages a multi-branch structure and dynamic weight fusion mechanism to enhance feature representation while achieving highly robust modeling of occlusion, pose variation, and scale differences. Moreover, the module is lightweight and efficient, offering strong potential for embedded deployment, and is particularly well suited for end-to-end, real-time, and fine-grained facial detection of chickens in precision farming scenarios.

#### 2.3.3. PEMD Module

**Figure 6:**
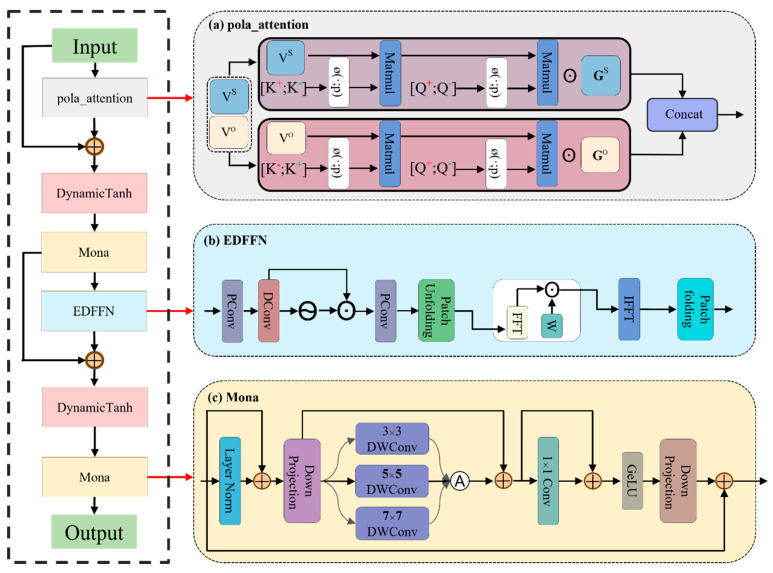
Structure of the PEMD module, subfigure (**a**) PolaAttention module that captures global context in polar coordinates with linear complexity, subfigure (**b**) EDFFN module that compensates high-frequency details through enhanced feedforward operations, and subfigure (**c**) Mona module that performs multi-scale fusion to aggregate features across scales.

Given the multiple performance bottlenecks exposed by the native AIFI architecture of RT-DETR in the context of chicken face detection, namely the exponential O(N^2^d) complexity of multi-head attention in high-dimensional sequence interactions and the over-smoothed Softmax response [23], the spatial blind spots and inefficiency in high-frequency information restoration of the standard FFN (OC2N) during channel mapping [25], the collapse of parallel throughput caused by the dependence of LayerNorm on μ and σ^2^ statistics [24], and the insufficient multi-scale coordination that results in global–local disharmony [22], we propose the PEMD composite module, which holds both theoretical and practical significance. As shown in Figure 6, this module is composed of four subsystems—PolaAttention, DynamicTanh, EDFFN, and Mona—systematically reshaping attention, normalization, and feed-forward networks, while achieving global recasting, lightweight normalization, high-frequency compensation, and multi-scale juxtaposition through multi-scale spatial fusion, thereby forming a four-stage closed loop.

During a single forward pass of this module, the input feature map $X\in R^{B\times C\times H\times W}$ is first flattened into [B, N, C] (where N = H × W) and then processed by Polar Coordinate Linear Attention (PolaAttention) to recast the global information. Learnable radial and angular projections replace the conventional Softmax, reducing the computational complexity of attention from O(N^2^d) to a linear level, while retaining sensitivity to subtle signals, thereby generating:

(8) $$Z_{1}=X+Drop_{1}\left(PolaAttention\left(reshape\left(X\right)\right)\right)$$

Subsequently, to eliminate dependence on batch statistics and enhance parallel throughput, the module adopts parameterized tanh normalization (DynamicTanh). By applying learnable scaling factors and offsets within the channel dimension, it performs smooth correction, significantly accelerating the normalization process and stabilizing the output distribution. Thus, we obtain:

(9) $$Z_{2}=DyT_{1}\left(Z_{1}\right)$$

After completing the lightweight normalization, the Mona submodule deploys multiple pooling and convolutional branches with different receptive fields in parallel, and dynamically assigns fusion weights based on the input features, achieving a coordinated aggregation of local and global information, outputting:

(10) $$Z_{3}=Mona_{1}\left(Z_{2}\right)$$

Subsequently, to recover high-frequency details that the standard FFN struggles to capture, an Efficient Dynamic Feedforward Network (EDFFN) was designed. It compensates for spatial blind spots through channel expansion, nonlinear activation, and multi-kernel dynamic convolution, and integrates residual connections with stochastic dropout to restore high-frequency information under near-linear complexity, balancing efficiency and detail reconstruction:

(11) $$Z_{4}=Z_{3}+Drop_{2}\left(EDFFN\left(Z_{3}\right)\right)$$

Finally, the module applies DynamicTanh again for a second normalization and, through Mona, achieves multi-scale feature adaptation with minimal parameter overhead, enhancing the perception of complex-shaped targets and producing the output feature map.

(12) $$Y=Mona_{2}\left(DyT_{2}\left(Z_{4}\right)\right)$$

Overall, the PEMD synthesis module systematically reshapes RT-DETR’s attention, normalization, and feedforward network structures through a four-stage closed loop that sequentially performs global re-casting, lightweight normalization, high-frequency recovery, and multi-scale juxtaposition, while embedding a multi-scale spatial fusion mechanism. Seamlessly integrated into the encoder, this module maintains the end-to-end detection pipeline and provides a robust structural foundation for achieving real-time responsiveness and high-precision performance in subsequent chicken face detection tasks under severe occlusion, variable poses, and complex lighting conditions.

#### 2.3.4. Matchability-Aware Loss Function

In this study, we replaced the original Varifocal Loss in the classification branch of the real-time chicken face detection model CF-DETR with the Matchability Aware Loss (MAL), in order to not only maintain sufficient optimization for high-quality matches but also to significantly enhance the model’s responsiveness to low-quality matches. The original Varifocal Loss can be expressed as:

(13) $$L_{VFL}\left(p,q\right)=\left\{\begin{array}{l} -q\left(q\log p+\left(1-q\right)\log\left(1-p\right)\right),q>0,\\ -\partial p^{\gamma }\log\left(1-p\right),q=0, \end{array}\right\}$$

In the equation, $p\in \left[0,1\right]$ represents the predicted foreground confidence by the model, $q\in \left[0,1\right]$ denotes the IoU (overlap) between the predicted box and the ground truth box, ∂ is used to balance the ratio of positive and negative samples, and γ controls the model’s focus between easy and hard samples. Since Varifocal Loss produces small gradients for matches with low IoU, it struggles to drive CF-DETR to sufficiently adjust for these critical but subtle feature errors, thereby limiting the precise localization of fine chicken face regions. To address this, we adopt the Matchability Aware Loss, expressed as follows:

(14) $$L_{MAL}\left(p,q,y\right)=\left\{\begin{array}{l} -q^{\gamma }\log p+\left(1-q^{\gamma }\right)\log\left(1-p\right),y=1,\\ -p^{\gamma }\log\left(1-p\right),y=0, \end{array}\right\}$$

Here $y\in \left[0,1\right]$ denotes the binary ground-truth label (positive when y=1, negative when y=0); the remaining symbols have the same meanings as in Equation (13). By replacing the positive sample label from q to $q^{\lambda }$, MAL not only eliminates the need to tune the hyperparameter ∂ but also imposes stronger penalties on low–IoU matches as the confidence p increases, effectively enhancing the model’s learning signal for matches that are easily overlooked.

In the CF-DETR model, we only need to replace the loss function in the classification branch from Varifocal Loss to Matchability Aware Loss, while the regression and matching processes remain entirely consistent with the original framework. Specifically, the overall loss function is adjusted as follows:

(15) $$L=\lambda_{cls}L_{MAL}\left(p,q,y\right)+\lambda_{l_{1}}\left\|b-\right.{\left.b^{*}\right\|}_{1}+\lambda_{GIoU}\left(1-GIoU\left(b,b^{*}\right)\right)$$

Through this simple substitution, CF-DETR not only retains the original advantages of one-to-one matching but also significantly strengthens the penalty on low-quality matches, thereby potentially improving the localization accuracy of subtle chicken face features and accelerating model convergence.

### 2.4. Experimental Environment and Parameter Settings

All experiments in this study were conducted on the Ubuntu 20.04 Linux operating system. The hardware platform utilized an NVIDIA GeForce RTX 3090 GPU with 24 GB of memory (NVIDIA Corporation, Santa Clara, CA, USA), providing powerful parallel computing capabilities sufficient to meet the computational demands of deep learning training. The software environment included the PyTorch 1.13.1 deep learning framework, the CUDA 11.6 acceleration platform, and Python 3.8.0 programming language. During model training, the AdamW optimizer was employed, with key hyperparameters such as learning rate, momentum, and regularization terms appropriately configured according to the task characteristics. All input images were resized to a uniform resolution of 640 × 640 during training and validation to ensure the model learns features at a fixed scale. The main training configurations are summarized in Table 2.

### 2.5. Evaluation Metrics

To comprehensively and multidimensionally assess the performance of the proposed model, this study established an evaluation framework encompassing five aspects: detection accuracy, object detection capability, overall performance, real-time capability, and model complexity. This framework includes core metrics such as Precision, Recall, F_1_-Score, mean Average Precision (mAP@0.5, mAP@0.5:0.95), frames per second (FPS), as well as the number of parameters (Parameters) and floating-point operations (FLOPs), enabling a quantitative and systematic analysis of the model’s performance across various application scenarios. The specific definitions are as follows:

(16) $$\mathrm{Pr}ecision=\frac{TP}{TP+FP}$$

(17) Recall=TPTP+FN

(18) F1−Score=2×precision×RecallPrecision+Recall

Here, TP, FP, and FN represent the numbers of true positive, false positive, and false negative samples, respectively.

(19) $$AP=\int_{0}^{1}P\left(r\right)dr$$

(20) $$mAP_{0.5}=\frac{1}{N}\sum_{i=1}^{N}AP_{i}\left(IoU=0.5\right)$$

(21) $$mAP_{0.5:0.95}=\frac{1}{N}\sum_{t=0.50:0.05:0.95}\left[\frac{1}{N}\sum_{i=1}^{N}AP_{i}\left(IoU=t\right)\right]$$

Here, N denotes the total number of classes, and IoU (Intersection over Union) measures the degree of overlap between the predicted and ground-truth boxes.

(22) $$FPS=\frac{1000ms}{T_{\mathrm{pre}}+T_{\inf}+T_{NMS}}$$

$T_{pre}$ represents the average time consumed during the preprocessing stage, $T_{\inf}$ represents the average time during the model inference stage, and NMS denotes the average time in the post-processing (non-maximum suppression) stage.

Parameters: the total number of trainable parameters (in millions, M); FLOPs: the number of floating-point operations required for a single forward pass (in billions, G).

## 3. Results

### 3.1. Backbone Comparison Experiment

To verify the effectiveness of the DIDC module, we compared four backbones under the same experimental settings: the original RT-DETR, a backbone based on the C2f structure, a backbone based on C2f integrated with the PKI module, and a backbone based on DIDC, i.e., C2f_DCMB (denoted as DIDC(C2f) in the table). Regarding lightweight design and embedded deployment requirements, the DIDC module employs three parallel depthwise separable convolution branches (square, horizontal stripe, and vertical stripe) along with a global context-based dynamic weight generation mechanism, reducing the model parameters from 19.9 M to 13.2 M and FLOPs from 56.9 G to 43.5 G, while maintaining an inference speed of 73.8 FPS, indicating that even with further reduction in computational cost, the DIDC module does not noticeably affect real-time performance and better meets the requirements for subsequent embedded deployment.

From the perspective of feature extraction capability, the DIDC module leverages multi-branch convolutions covering different directions and receptive fields and adaptively fuses the outputs of each branch with global semantic signals, effectively enhancing the model’s adaptability to chicken face regions of varying scales and complex shapes. As shown in Table 3, after introducing the DIDC module, Precision increased from 93.8% to 96.2%, F_1_-Score from 92.9% to 94.3%, mAP50 from 95.4% to 96.3%, and mAP50:95 from 60.2% to 61.4%, surpassing all other comparison models on multiple key metrics and fully validating the design advantages of the DIDC module in multi-scale spatial feature extraction and dynamic fusion.

### 3.2. Classification Loss Comparison Experiment

To meet the dual requirements of high accuracy and fast convergence in chicken face detection, this study compared four classification loss functions: VariFocal Loss (VFL) [30], Slide Loss [31], EMASlide Loss [32], and MAL [26] to determine the optimal solution.

**Figure 7:**
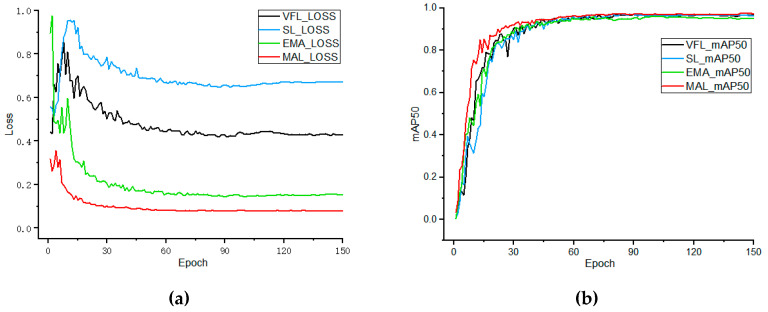
(**a**) Comparison of the four classification loss functions; (**b**) changes in mAP50 after introducing the four different classification loss functions.

As shown in Table 4, in the comparison experiment of the four classification loss functions, MAL outperformed all others across all key detection metrics. Specifically, MAL achieved 95.2% Precision, 93.1% Recall, and 94.1% F_1_-Score, significantly exceeding VFL’s 93.8%, 92.1%, and 92.9%; meanwhile, MAL reached 96.3% mAP50 and 61.1% mAP50:95, representing improvements of 0.9 and 0.9 percentage points over VFL, respectively, and also outperformed Slide Loss (95.6%/60.7%) and EMASlide Loss (96.0%/59.3%). The mAP50 curves corresponding to each loss function in Figure 7b clearly show that the MAL curve remains at the highest level and maintains the smallest decline during later iterations, further demonstrating its advantages in accuracy and robustness.

From the perspective of training convergence speed, the loss curves shown in Figure 7a indicate that MAL reaches a convergence plateau at around 31 epochs, whereas VFL and Slide Loss require 37 and 39 epochs, respectively. Although EMASlide Loss also converges at approximately 31 epochs, its final mAP50 and mAP50:95 still lag behind MAL. Considering MAL’s leading performance in Precision, Recall, F_1_, and mAP metrics as shown in Table 4, along with the fastest convergence speed and lowest training oscillation in Figure 7a, it is evident that MAL not only significantly shortens the training cycle but also improves detection performance. Based on this rigorous data comparison and analysis, MAL was ultimately selected as the classification loss function for the CF-DETR model.

### 3.3. Ablation Experiment

To verify the effectiveness of the systematic structural optimization proposed in this study within the RT-DETR framework—focusing on three key aspects: feature extraction (DIDC), information encoding (PEMD), and loss design (MAL)—we conducted ablation experiments based on RT-DETR under the same experimental settings. The experiments considered not only commonly used detection accuracy metrics (mAP50, mAP50:95, Precision, Recall, F_1_) but also included model parameters, FLOPs, and inference speed, enabling a comprehensive evaluation from both detection performance and embedded deployment feasibility perspectives.

As shown in Table 5, introducing the DIDC module alone reduces the model parameters from 19.9 M to 13.2 M (a 33.7% decrease) and FLOPs from 56.9 G to 43.5 G (a 23.5% decrease), while maintaining an inference speed of 73.8 FPS, demonstrating its suitability for embedded deployment. In addition, its multi-scale convolutional branches and global context-based dynamic weight fusion enable finer extraction of local textures and edge information of chicken faces, increasing Precision to 96.2% (+2.4%) and mAP50:95 to 61.4% (+1.2%), while Recall fluctuates by no more than 0.5% (92.6%), validating its efficient feature extraction capability. Although the PEMD encoding module increases the model parameters to 20.0 M and FLOPs to 57.2 G, its PolarAttention reconstructs global dependencies in the polar coordinate domain with linear complexity, the Mona branch adaptively fuses multi-scale pooling and convolutional branches, and EDFFN with DynamicTanh compensates high-frequency details and stabilizes feature distributions, resulting in Recall increasing to 93.6% (+1.5%) and mAP50:95 rising to 61.2% (+1.0%), fully demonstrating its advantage in global–local collaborative perception for small and occluded chicken-face targets.

When the DIDC and PEMD modules are applied simultaneously, the model further balances accuracy and speed while maintaining efficient computation, achieving 94.9% Precision and 93.1% Recall, with mAP50 increasing to 96.7% and mAP50:95 rising to 62.0%, while parameters remain at 13.3 M, FLOPs at 43.8 G, and the frame rate improves to 80.6 FPS. On this basis, by integrating the Matchability Aware Loss (MAL) into CF-DETR, the model’s learning signal becomes more focused on low-quality matched samples, resulting in a more balanced performance improvement. The final CF-DETR achieves 95.5% Precision, 94.6% Recall, and 95.1% F_1_-Score, representing a 1.1 percentage point improvement in F_1_ compared to the combination model without MAL; mAP50 reaches 96.9%, mAP50:95 further increases to 62.8%, while parameters and FLOPs remain at 13.3 M and 43.8 G, and the frame rate stabilizes at 81.4 FPS.

**Figure 8:**
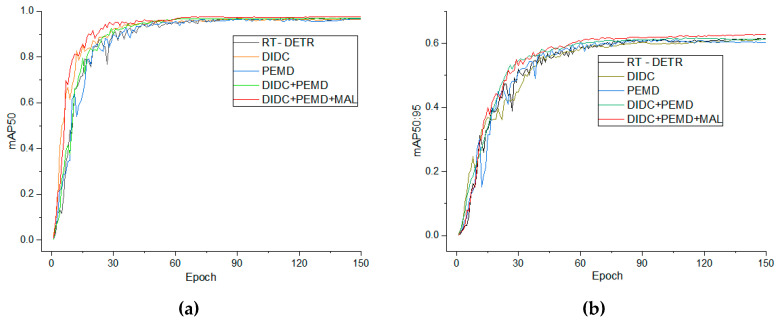
Comparison of mAP50 and mAP50:95 for each scheme during 150 training epochs in the ablation experiment. Subfigure (**a**) shows the mAP50 curves over training epochs; subfigure (**b**) shows the mAP50:95 curves over training epochs.

The training curves in Figure 8 clearly demonstrate the combined advantages of the DIDC–PEMD–MAL configuration in convergence speed, training stability, and final plateau height. First, the DIDC module enhances the representation of fine-grained and direction-sensitive features, promoting the quality of candidate boxes in the early training stages and accelerating the rise of mAP. Second, PEMD, through polar-coordinate attention, distribution stabilization, and high-frequency detail compensation, effectively mitigates performance decline in the middle and later stages, resulting in a smoother convergence curve and a higher plateau. Finally, MAL strengthens the gradient response to low-quality matched samples through its match-driven loss design, significantly suppressing training oscillations and improving consistency in localization and confidence. Therefore, the steeper rise, earlier stabilization, and higher plateau shown in the figure are not isolated phenomena but a direct reflection of the three modules complementing each other during training, indicating that DIDC–PEMD–MAL indeed outperforms other comparison configurations in terms of convergence and generalization capability.

This combined configuration outperforms any single- or dual-module combination in both detection accuracy and deployment efficiency, fully validating the synergistic optimization of DIDC, PEMD, and MAL in structural design, information encoding, and loss modeling. Overall, the modules complement each other across different task levels, providing robust support for high-performance, low-resource embedded real-time chicken face detection.

### 3.4. Visualization Analysis

**Figure 9:**
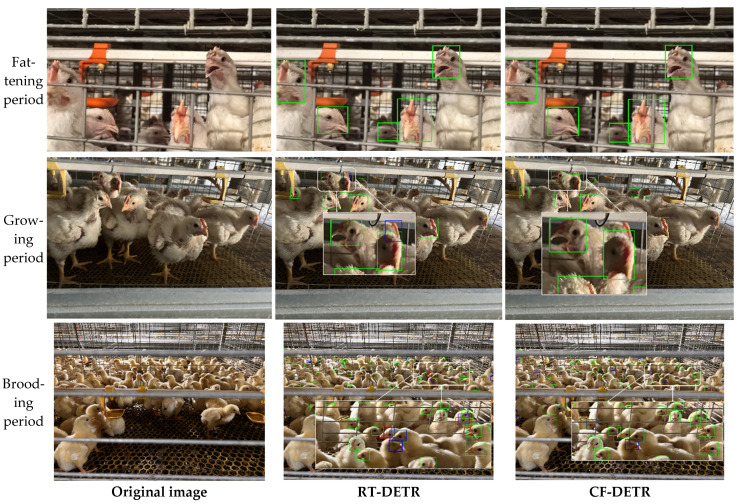
Detection comparison between RT-DETR and CF-DETR under complex samples from the fattening, growing, and brooding periods. The left column shows the original images, the middle column shows RT-DETR detection results, and the right column shows CF-DETR detection results. Box colors indicate the following: green for true positives (TP); blue for false positives (FP); and red for false negatives (FN). The legend also annotates the number of each type of box for quantitative comparison.

To intuitively evaluate the detection robustness and interpretability of the baseline RT-DETR and the proposed CF-DETR in complex farming environments across different growth stages, we selected representative samples from the fattening, growing, and brooding periods for visual comparison. As shown in Figure 9, during the fattening period, the targets are relatively large and feature distinctions are high, allowing both models to perform stable detection with almost no false positives or false negatives. In the growing period, as target size decreases and neighborhood interference increases, the baseline model produces false positives due to the similarity of adjacent head features, whereas CF-DETR maintains stable detection results, showing no missed detections and significantly fewer false positives. In the most challenging brooding period, targets are smaller, stocking density is higher, and occlusion is severe, causing the baseline model to exhibit both missed detections and false positives. Missed detections result from feature weakening and lost candidate boxes, while false positives mainly arise from background textures and neighboring interference. In contrast, CF-DETR maintains zero missed detections during this stage, with fewer false positives than the baseline RT-DETR, fully demonstrating its significant advantages in feature extraction, occlusion handling, and interference suppression.

**Figure 10:**
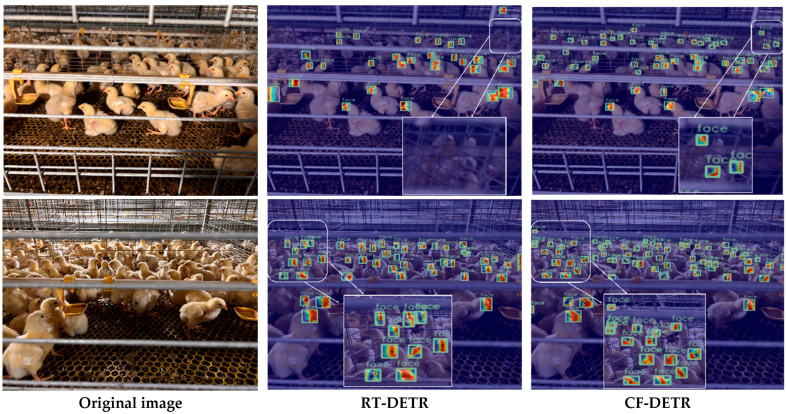
Quantitative comparison of Grad-CAM heatmaps for brooding-period samples (RT-DETR vs. CF-DETR).

To further reveal the internal mechanism behind performance differences in the brooding period, we conducted heatmap visualization analysis on brooding-period samples. As shown in Figure 10, compared with the baseline model, CF-DETR exhibits clear and stable activation responses for more targets during the brooding period, whereas the baseline model lacks identifiable activation signals on several true targets. Further observation shows that the unactivated targets in the heatmaps highly coincide with the detector’s missed detections, indicating that the absence of internal responses is an important cause of missed detections. From this, it can be concluded that heatmaps, as a form of explainable evidence, support that CF-DETR possesses stronger feature response capability and higher recall in small-scale, high-density, and heavily occluded brooding-period scenarios, providing an intuitive visual confirmation of the performance improvement of our method. It should be emphasized that heatmaps are an interpretability tool rather than direct detection outputs; this section aims to provide an intuitive understanding of model behavior and supplementary evidence.

### 3.5. Comparison with Other Models

To comprehensively and objectively evaluate the practical performance of the proposed CF-DETR in chicken face detection, we conducted a horizontal comparison under the same dataset and evaluation criteria with representative single-stage detectors (YOLOv10m, YOLOv11m) [33,34], anchor-based single-stage detector (TOOD) [35], two-stage detector (Faster R-CNN) [36], and Transformer-based detectors (DETR, RT-DETR, RT-DETR-r34). The comparison metrics include detection accuracy (Precision, Recall, mAP@0.5, mAP@0.5:0.95), real-time performance (FPS), and model complexity (Parameters, FLOPs), aiming to reveal the strengths and limitations of each method across the three dimensions of accuracy, speed, and resource efficiency. The results are shown in Table 6.

From the data, the models exhibit clear performance differentiation. YOLOv11m slightly leads in Precision (P = 95.8%), but its inference speed is only 31.6 FPS, indicating that although post-processing reduces false positives, the heavy computation and post-processing overhead limit its real-time performance for this task. Similarly, YOLOv10m achieves a near-leading mAP50 (96.5%) but an FPS of only 37.7, showing that single-stage networks on this dataset are still affected by NMS post-processing and model complexity. Two-stage and improved two-stage methods (Faster R-CNN, TOOD) show moderate performance in some accuracy metrics but at the cost of substantially higher parameters and FLOPs (e.g., Faster R-CNN parameters ≈ 41.3 M, FLOPs ≈ 208 G), resulting in low FPS (below 34), which is unfavorable for embedded and real-time applications. The original DETR performs poorly in this high-density scenario (mAP50 = 88.6%), indicating that standard Transformer-only architecture struggles with tiny-scale and occluded chicken faces without targeted local-scale enhancement. RT-DETR provides a good speed–accuracy trade-off (mAP50 = 95.4%, FPS = 74.1), but compared with the proposed CF-DETR, it still lags in Recall (RT-DETR R = 92.1% vs. CF-DETR R = 94.6%) and overall mAP (95.4% vs. 96.9%). The comparison results indicate that CF-DETR achieves a better balance between accuracy and efficiency, attaining the highest mAP50 (96.9%) and mAP50:95 (62.8%) while maintaining the lowest parameter count (13.3 M) and computational cost (43.8 G), and leading all compared models with an inference speed of 81.4 FPS, demonstrating the practical benefits of the synergistic optimization across backbone (DIDC), encoder (PEMD), and loss design (MAL) in this study.

In summary, although some YOLO-series models show advantages in individual metrics (such as Precision or single-point mAP), their disadvantages in real-time performance or computational resource requirements limit their feasibility for practical deployment. Two-stage methods and pure DETR-based approaches are also constrained in this task due to model complexity or lack of adaptation mechanisms. In contrast, CF-DETR, through lightweight structural design and targeted encoding and loss strategies, demonstrates the most balanced and superior performance in chicken face detection under high-density and heavily occluded real-world scenarios.

## 4. Discussion

This study constructed a chicken-face detection dataset covering the entire growth cycle of chickens, from the chick stage to the fattening stage, encompassing significant variations in facial size and texture across different stages (see Figure 1). During the early chick stage, the facial region is extremely small, features are difficult to discern, and high stocking density results in severe occlusion. In the growing stage, the facial area increases but neighboring interference also rises. In the fattening stage, the facial region is largest, and features are most stable. Such high-density, fine-grained detection scenarios pose significant challenges to detectors [10,11]. To address this, we selected RT-DETR as the starting point. RT-DETR offers advantages in end-to-end detection and the removal of NMS, while achieving a balance between accuracy and speed through a hybrid convolutional backbone and an efficient encoder. However, the native RT-DETR structure exhibits shortcomings in chicken face scenarios. The ResNet backbone has limited capability to express subtle texture differences [37]. The AIFI module provides sufficient global modeling ability but insufficient local detail recovery [25], and attention based on normalization and Softmax introduces parallelism and efficiency bottlenecks [23,24], while lacking juxtaposed structural interaction and multi-receptive-field collaboration [22]. These factors further limit the effectiveness of the matching mechanism optimization and training convergence speed [26], and previous work has pointed out that the DETR framework struggles to capture fine-grained information [38]. Therefore, this study proposes a systematic improvement scheme addressing the three major bottlenecks of RT-DETR: feature extraction, information encoding, and loss design.

First, at the backbone network level, we designed the DIDC module, which employs multi-branch depthwise separable convolutions (including stripe convolutions in different orientations) combined with global semantic dynamic fusion, effectively enhancing the perception of chicken faces of varying scales and complex shapes. As shown in Table 3, after introducing DIDC, both Precision and mAP improved significantly (Precision increased from 93.8% to 96.2%, mAP50 increased from 95.4% to 96.3%), while the model parameters decreased by over one-third, indicating that the multi-branch convolutions preserve fine-grained texture features while improving efficiency. Second, at the encoder level, we proposed the PEMD module, which incorporates polar-coordinate attention and a high-frequency detail compensation mechanism. Polar-coordinate attention enables global context capture with linear complexity, while the Mona multi-scale fusion branch together with EDFFN and DynamicTanh compensates for high-frequency information. In the ablation experiments shown in Table 5, introducing PEMD alone increased Recall by 1.5 percentage points (from 92.1% to 93.6%) and raised mAP50:95 from 60.2% to 61.2%. These results indicate that PEMD enhances the model’s global–local collaborative perception under occlusion and background interference, thereby improving detection robustness and overall performance. Furthermore, the proposed Matchability Aware Loss (MAL) makes the training signal more sensitive to hard samples through dynamic label weighting. Comparative experiments in Table 4 demonstrate that MAL outperforms traditional losses such as VariFocal Loss in both precision and mAP (Precision increased from 93.8% to 95.2%, mAP50 increased from 95.4% to 96.3%), and as shown in Figure 7, it achieves faster convergence and minimal training oscillation. Specifically, MAL converges in approximately 31 epochs, whereas VariFocal Loss requires 37 epochs; as illustrated in the loss and mAP curves in Figure 7a, MAL consistently maintains the highest level, further validating its effectiveness in training stability and performance improvement.

Based on the comprehensive ablation results (Table 5) and the convergence curves (Figure 7 and Figure 8), the synergistic effects of the three mechanisms are clearly evident. DIDC accelerated the early-stage rise of mAP during training, PEMD mitigated the late-stage performance decline, and MAL smoothed oscillations during training, resulting in steeper convergence curves and higher performance plateaus. In the visualization analysis of representative samples, CF-DETR exhibited more robust detection performance compared to the baseline RT-DETR. For growing-stage samples, RT-DETR produced false positives due to similar neighboring head features, whereas CF-DETR showed almost zero missed and false detections. In the most challenging chick-stage samples, RT-DETR exhibited obvious missed and false detections (missed detections resulting from weakened features and false detections caused by background interference), while CF-DETR maintained zero missed detections and significantly reduced false detections. Grad-CAM heatmaps (Figure 10) further confirmed this, showing that CF-DETR generated clear and stable activation responses for more true chicken face targets, whereas RT-DETR lacked identifiable responses for some targets, and these weak-response regions highly corresponded to missed detection instances. This indicates that the modules in CF-DETR effectively enhance the model’s feature response capability for small-scale and occluded targets, providing visual evidence supporting the performance improvement. In comparison with other mainstream models (Table 6), CF-DETR achieved the highest mAP and real-time detection speed while maintaining the lowest parameter count and computational load, demonstrating the practical benefits of the design optimizations.

Although this study demonstrates significant advantages in chicken face detection across the full growth cycle, there are still two limitations to note. First, the data source is relatively homogeneous, covering only WOD168 white-feather broilers, which somewhat limits the model’s generalization ability to other breeds and different rearing conditions. Second, in chick-stage scenarios, although overall performance is markedly improved compared to the baseline model, CF-DETR still produces some false positives due to extremely small target sizes and frequent occlusion, as illustrated in Figure 9. To address these issues, future work will focus on expanding cross-breed and cross-scenario data validation, and incorporating targeted small-object enhancement and temporal information modeling in the model design to further improve robustness and generalizability. Moreover, considering potential on-farm deployment, future research will further investigate hardware adaptability, energy efficiency, and seamless integration with existing farm management systems to ensure practical applicability in real-world poultry farming environments. Finally, as the dataset used in this study is currently under collaboration with relevant enterprises and has not been publicly released, future plans include making the dataset available upon project completion to facilitate subsequent research.

In summary, the experimental analysis demonstrates that the proposed multi-branch convolutional feature extraction module (DIDC), closed-loop encoder (PEMD), and match aware loss (MAL) effectively address the adaptation bottlenecks of RT-DETR in chicken face detection, significantly enhancing performance in high-density and heavily occluded complex scenarios. Each improvement mechanism has been thoroughly validated through ablation and visualization experiments: the DIDC module enriches spatial multi-scale feature representation, the PEMD module compensates high-frequency details and strengthens global–local fusion, and the MAL module focuses the learning signal on low-quality matched samples, improving recall and the consistency of localization confidence. The proposed modules exhibit complementarity, aligning with recent Transformer improvement strategies that emphasize stronger feature representation and more complete multi-scale information aggregation to enhance the model’s adaptability to occlusion, background interference, and scale variation [37,39]. Therefore, CF-DETR demonstrates clear advantages in accurately and efficiently detecting broiler chicken faces, providing a solid foundation for real-time intelligent poultry monitoring.

## 5. Conclusions

This study addresses the chicken face detection task in high-density farming scenarios and proposes a systematic improvement scheme based on the RT-DETR framework. By introducing the multi-branch convolutional feature extraction module (DIDC), the polar-coordinate closed-loop encoder (PEMD), and the match aware loss (MAL), we constructed a lightweight and efficient end-to-end detection model, CF-DETR. Experiments demonstrate that CF-DETR achieves the optimal balance between accuracy and real-time performance: while maintaining only 13.3 M parameters and 43.8 G FLOPs, it reaches 96.9% mAP50 and 94.6% recall, with an inference speed of 81.4 FPS, outperforming other comparative models. Compared to the baseline RT-DETR, CF-DETR significantly enhances the detection capability for small-scale and occluded targets and accelerates training convergence. The structural optimizations and loss strategies designed in this study not only effectively address the specific demands of chicken face detection but also provide feasible technical approaches and theoretical support for future precise and intelligent livestock monitoring.

## Figures and Tables

**Table 1:** Statistics of training, validation, and test splits before and after augmentation.

Statistic	Test Set	Validation Set	Original Training Set	Augmented Training Set	Total Training Set	Pre-Augmentation Total	Post-Augmentation Total
Number of Images	100	100	800	120	920	1000	1120

**Table 2:** Experimental parameter settings.

Hyperparameter Selection	Setting
Input Image Size	640 × 640
Optimizer	AdamW
Initial Learning Rate	0.0001
Momentum	0.9
Weight Decay	0.0001
Batch Size	8
Epoch	150

**Table 3:** Comparison results of different backbone networks.

Model	P (%)	R (%)	F_1_ (%)	mAP50 (%)	mAP50:95 (%)	Param (M)	FLOPs (G)	FPS
RT-DETR	93.8	92.1	92.9	95.4	60.2	19.9	56.9	74.1
C2f	96.3	91.3	93.7	95.6	58.3	15.4	52.6	68
PKI (C2f)	95.9	92.5	94.2	96.5	57.8	17.7	57.2	64.5
DIDC (C2f)	96.2	92.6	94.3	96.3	61.4	13.2	43.5	73.8

**Table 4:** Classification loss replacement results.

Model	P (%)	R (%)	F_1_ (%)	mAP50 (%)	mAP50:95 (%)
VariFocal LOSS	93.8	92.1	92.9	95.4	60.2
Slide Loss	93.3	92.5	92	95.6	60.7
EMASlide Loss	93.6	91.3	93.2	96	59.3
MAL	95.2	93.1	94.1	96.3	61.1

**Table 5:** Ablation experiment results.

Model	P (%)	R (%)	F_1_ (%)	mAP50 (%)	mAP50:95 (%)	Param (M)	FLOPs (G)	FPS
RT-DETR(Base)	93.8	92.1	92.9	95.4	60.2	19.9	56.9	74.1
DIDC	96.2	92.6	94.3	96.3	61.4	**13.2**	43.5	73.8
PEMD	94.2	93.6	93.9	96.3	61.2	20	57.2	72
MAL	95.2	93.1	94.1	96.3	61.1	19.9	56.9	76
DIDC + PEMD	94.9	93.1	94	96.7	62	13.3	43.8	80.6
CF-DETR (DIDC + PEMD + MAL)	95.5	**94.6**	**95.1**	**96.9**	**62.8**	13.3	**43.8**	**81.4**

**Table 6:** Comparison results with other models.

Model	P (%)	R (%)	mAP50 (%)	mAP50:95 (%)	Param (M)	FLOPs (G)	FPS
YOLOv10m	95.6	90.4	96.5	61.2	16.4	64	37.7
YOLOv11m	95.8	93.1	95.8	61.6	20.1	68.5	31.6
Tood	91.8	86.9	92.3	51.4	32	168	21
Faster_rcnn	94.2	87.1	93.4	54.6	41.3	208	34
DETR	92.9	85.5	88.6	43.6	41.5	81.6	31.4
RT-DETR	93.8	92.1	95.4	60.2	19.9	56.9	74.1
RT-DETR-r34	95.6	93.4	96.1	59.8	31.1	88.8	71.9
CF-DETR	95.5	94.6	96.9	62.8	13.3	43.8	81.4

## Data Availability

The data provided in this study are available upon request from the corresponding author.

## References

[1] Kryger K.N., Thomsen K.A., Whyte M.A., Dissing M. (2010). Smallholder Poultry Production–Livelihoods, Food Security and Sociocultural Significance. FAO Smallhold. Poult. Prod. Pap..

[2] Sheheli S., Hasan M.M., Dev D.S., Hasan M.M. (2024). Livelihood Improvement of Broiler Farmers in Bhaluka of Mymensingh District, Bangladesh. J. Bangladesh Agric. Univ..

[3] Satapathy D., Sharma A., Paswan J.K., Sarkar S., Varun T.K. (2017). Economic Broiler Farming: Scope and Limitations. Indian Farmers.

[4] Olejnik K., Popiela E., Opaliński S. (2022). Emerging Precision Management Methods in Poultry Sector. Agriculture.

[5] Yang X., Bist R.B., Paneru B., Chai L. (2024). Deep Learning Methods for Tracking the Locomotion of Individual Chickens. Animals.

[6] Al-Marzooqi W. (2024). Modern Technology and Traditional Husbandry of Broiler Farming.

[7] Doornweerd J.E., Veerkamp R.F., de Klerk B., van der Sluis M., Bouwman A.C., Ellen E.D., Kootstra G. (2024). Tracking Individual Broilers on Video in Terms of Time and Distance. Poult. Sci..

[8] Gao B., Guo Y., Zheng P., Yang K., Chen C. (2025). A Novel Lightweight Framework for Non-Contact Broiler Face Identification in Intensive Farming. Sensors.

[9] Kern D., Schiele T., Klauck U., Ingabire W. (2025). Towards Automated Chicken Monitoring: Dataset and Machine Learning Methods for Visual, Noninvasive Reidentification. Animals.

[10] Ma X., Lu X., Huang Y., Li L. (2022). An Advanced Chicken Face Detection Network Based on GAN and MAE. Animals.

[11] Guo Y., Wu Z., You B., Chen L., Zhao J., Li X. (2025). YOLO-SDD: An Effective Single-Class Detection Method for Dense Livestock Production. Animals.

[12] Li G., Huang Y., Chen Z., Zhao Y. (2021). Practices and Applications of Convolutional Neural Network-Based Computer Vision Systems in Animal Farming: A Review. Sensors.

[13] Zhang H., Li H., Sun G., Yang F. (2025). MDA-DETR: Enhancing Offending Animal Detection with Multi-Channel Attention and Multi-Scale Feature Aggregation. Animals.

[14] Alif M.A.R., Hussain M. (2024). YOLOv1 to YOLOv10: A Comprehensive Review of YOLO Variants and Their Application in the Agricultural Domain. arXiv.

[15] Wu Z., Yang J., Zhang H., Fang C. (2025). Enhanced Methodology and Experimental Research for Caged Chicken Counting Based on YOLOv8. Animals.

[16] Nasiri A., Amirivojdan A., Zhao Y., Gan H. (2023). Estimating the Feeding Time of Individual Broilers via Convolutional Neural Network and Image Processing. Animals.

[17] Zhang X., Zhu R., Zheng W., Chen C. (2024). Detection of Leg Diseases in Broiler Chickens Based on Improved YOLOv8 X-Ray Images. IEEE Access.

[18] Syafaah L., Faruq A., Setyawan N. (2024). Sick and Dead Chicken Detection System Based on YOLO Algorithm. Ingénierie Systèmes Inf..

[19] Carion N., Massa F., Synnaeve G., Usunier N., Kirillov A., Zagoruyko S. End-to-End Object Detection with Transformers. Proceedings of the European Conference on Computer Vision—ECCV 2020.

[20] Zhao Y., Lv W., Xu S., Wei J., Wang G., Dang Q., Liu Y., Chen J. DETRs Beat YOLOs on Real-Time Object Detection. Proceedings of the 2024 IEEE/CVF Conference on Computer Vision and Pattern Recognition (CVPR).

[21] Lv Z., Dong S., Xia Z., He J., Zhang J. (2025). Enhanced Real-Time Detection Transformer (RT-DETR) for Robotic Inspection of Underwater Bridge Pier Cracks. Autom. Constr..

[22] Yin D., Hu L., Li B., Zhang Y., Yang X. 5%>100%: Breaking Performance Shackles of Full Fine-Tuning on Visual Recognition Tasks. Proceedings of the 2025 IEEE/CVF Conference on Computer Vision and Pattern Recognition (CVPR).

[23] Meng W., Luo Y., Li X., Jiang D., Zhang Z. (2025). PolaFormer: Polarity-Aware Linear Attention for Vision Transformers. arXiv.

[24] Zhu J., Chen X., He K., LeCun Y., Liu Z. Transformers without Normalization. Proceedings of the 2025 IEEE/CVF Conference on Computer Vision and Pattern Recognition (CVPR).

[25] Kong L., Dong J., Tang J., Yang M.-H., Pan J. Efficient Visual State Space Model for Image Deblurring. Proceedings of the 2025 IEEE/CVF Conference on Computer Vision and Pattern Recognition (CVPR).

[26] Huang S., Lu Z., Cun X., Yu Y., Zhou X., Shen X. DEIM: DETR with Improved Matching for Fast Convergence. Proceedings of the 2025 IEEE/CVF Conference on Computer Vision and Pattern Recognition (CVPR).

[27] Perez L., Wang J. (2017). The Effectiveness of Data Augmentation in Image Classification Using Deep Learning. arXiv.

[28] Leavline E.J., Singh D.A.A.G. (2013). Salt and Pepper Noise Detection and Removal in Gray Scale Images: An Experimental Analysis. IJSIP.

[29] Nie Z., Long T., Zhou Z. Research on Rotational Object Recognition Based on HSV Color Space and Gamma Correction. Proceedings of the 6th International Symposium on Advanced Technologies and Applications in the Internet of Things Kusatsu.

[30] Zhang H., Wang Y., Dayoub F., Sunderhauf N. VarifocalNet: An IoU-Aware Dense Object Detector. Proceedings of the 2021 IEEE/CVF Conference on Computer Vision and Pattern Recognition (CVPR).

[31] Yu Z., Huang H., Chen W., Su Y., Liu Y., Wang X. (2024). YOLO-FaceV2: A Scale and Occlusion Aware Face Detector. Pattern Recognit..

[32] Wu J., Zhao F., Yao G., Jin Z. (2025). FGA-YOLO: A one-stage and high-precision detector designed for fine-grained aircraft recognition. Neurocomputing.

[33] Khanam R., Hussain M. (2024). YOLOv11: An Overview of the Key Architectural Enhancements. arXiv.

[34] Wang A., Chen H., Liu L., Chen K., Lin Z., Han J., Ding G. (2024). YOLOv10: Real-Time End-to-End Object Detection. Adv. Neural Inf. Process. Syst..

[35] Feng C., Zhong Y., Gao Y., Scott M.R., Huang W. TOOD: Task-Aligned One-Stage Object Detection. Proceedings of the 2021 IEEE/CVF International Conference on Computer Vision (ICCV).

[36] Girshick R. Fast R-CNN. Proceedings of the IEEE International Conference on Computer Vision (ICCV).

[37] Ye Y., Sun Q., Cheng K., Shen X., Wang D. (2025). A Lightweight Mechanism for Vision-Transformer-Based Object Detection. Complex Intell. Syst..

[38] Huang J., Wang H. (2024). Small Object Detection by DETR via Information Augmentation and Adaptive Feature Fusion. arXiv.

[39] Cao X., Wang H., Wang X., Hu B. (2024). DFS-DETR: Detailed-Feature-Sensitive Detector for Small Object Detection in Aerial Images Using Transformer. Electronics.

